# Calcium-sensing receptors regulate cardiomyocyte Ca^2+ ^signaling via the sarcoplasmic reticulum-mitochondrion interface during hypoxia/reoxygenation

**DOI:** 10.1186/1423-0127-17-50

**Published:** 2010-06-17

**Authors:** Fang-hao Lu, Zhiliang Tian, Wei-hua Zhang, Ya-jun Zhao, Hu-lun Li, Huan Ren, Hui-shuang Zheng, Chong Liu, Guang-xia Hu, Ye Tian, Bao-feng Yang, Rui Wang, Chang-qing Xu

**Affiliations:** 1 Department of Pathophysiology, Harbin Medical University, Harbin 150086, China; 2 Department of Pediatrics, the second affiliated Hospital of Harbin Medical University, Harbin 150086, China; 3 Department of Neurobiology, Harbin Medical University, Harbin 150086, China; 4 Department of Immunology, Harbin Medical University, Harbin 150086, China; 5 Bio-pharmaceutical Key Laboratory of Heilongjiang Province, Harbin Medical University, Harbin 150086, China; 6 Department of Biology, Lakehead University, Thunder Bay, Ontario, P7B5E1, Canada

## Abstract

Communication between the SR (sarcoplasmic reticulum, SR) and mitochondria is important for cell survival and apoptosis. The SR supplies Ca^2+ ^directly to mitochondria via inositol 1,4,5-trisphosphate receptors (IP_3_Rs) at close contacts between the two organelles referred to as mitochondrion-associated ER membrane (MAM). Although it has been demonstrated that CaR (calcium sensing receptor) activation is involved in intracellular calcium overload during hypoxia/reoxygenation (H/Re), the role of CaR activation in the cardiomyocyte apoptotic pathway remains unclear. We postulated that CaR activation plays a role in the regulation of SR-mitochondrial inter-organelle Ca^2+ ^signaling, causing apoptosis during H/Re. To investigate the above hypothesis, cultured cardiomyocytes were subjected to H/Re. We examined the distribution of IP_3_Rs in cardiomyocytes via immunofluorescence and Western blotting and found that type 3 IP_3_Rs were located in the SR. [Ca^2+^]i, [Ca^2+^]_m _and [Ca^2+^]_SR _were determined using Fluo-4, x-rhod-1 and Fluo 5N, respectively, and the mitochondrial membrane potential was detected with JC-1 during reoxygenation using laser confocal microscopy. We found that activation of CaR reduced [Ca^2+^]_SR_, increased [Ca^2+^]_i _and [Ca^2+^]_m _and decreased the mitochondrial membrane potential during reoxygenation. We found that the activation of CaR caused the cleavage of BAP31, thus generating the pro-apoptotic p20 fragment, which induced the release of cytochrome *c *from mitochondria and the translocation of bak/bax to mitochondria. Taken together, these results reveal that CaR activation causes Ca^2+ ^release from the SR into the mitochondria through IP_3_Rs and induces cardiomyocyte apoptosis during hypoxia/reoxygenation.

## Background

The mitochondrion is a fundamental organelle that is intimately involved in many aspects of cellular physiology, such as energy production, free radical production, regulation of cytosolic Ca^2+ ^signaling pathways and apoptosis [1,2]. The mitochondrion also acts as a spatial Ca^2+ ^buffer that reduces cytosolic Ca^2+ ^overload and regulates Ca^2+^-dependent signaling in the cytosol. Mitochondrial Ca^2+ ^is taken up from the cytosol via a low-affinity Ca^2+ ^uniporter at mitochondrial membranes [3]. However, the intracellular Ca^2+ ^concentration ([Ca^2+^]i) is not high enough to initiate the uniporter under physiological conditions. Therefore, it has been postulated that activation of the inositol 1,4,5-trisphosphate receptors (IP_3_Rs) signaling pathway could release Ca^2+ ^from the sarcoplasmic reticulum (SR) to increase the microdomain Ca^2+ ^concentration ([Ca^2+^]) at focal contacts, known as mitochondria-associated ER membranes (MAM), between the SR and mitochondria, and then activate the uniporter. Recent studies have suggested that IP_3_Rs are highly compartmentalized at MAMs, providing direct mitochondrial Ca^2+ ^signaling. Cardiomyocytes contain an abundance of mitochondria, many of which are in close apposition to SR Ca^2+ ^release sites [4].

The SR is a multifunctional organelle that controls protein translation and Ca^2+ ^homeostasis. Under SR stress (e.g., SR Ca^2+ ^depletion), SR chaperone proteins such as Grp78 and Grp94 are up-regulated [5]. Prolonged SR stress will initiate apoptotic signals in the SR, including bax/bak-translocation to the SR to activate the release of Ca^2+ ^from the SR, cleavage and activation of procaspase 12 and BAP31, and Ire 1-mediated activation of apoptosis signal-regulating kinase 1 (ASK1)/c-Jun N-terminal kinase (JNK) [6].

The calcium-sensing receptor (CaR) is a member of the family of G protein-coupled receptors (GPCRs). One of the effects of CaR signal transduction is the activation of phospholipase C, which leads to the generation of the secondary messengers diacylglycerol (DAG) and inositol 1,4,5 trisphosphate (IP_3_). IP_3 _then mobilizes Ca^2+ ^from intracellular stores via the activation of specific IP_3 _receptors [7]. Wang et al. and Tfelt-Hansen et al. reported that CaR was functionally expressed in rat cardiac tissue and rat neonatal ventricular cardiomyocytes, respectively [8,9]. Later, Berra-Romani et al. showed that cardiac microvascular endothelial cells express a functional CaR [10]. Our group has demonstrated that CaR is involved in apoptosis in isolated adult rat hearts and in rat neonatal cardiomyocytes during ischemia/reperfusion [11]. Although it is known that CaR elevates the intracellular calcium concentration and then induces apoptosis, the in-depth mechanisms are still not known. The aim of this study was to investigate whether [Ca^2+^]_SR _would change with CaR activation in response to hypoxia/reoxygenation in cardiomyocytes. We specifically focused on the relationship between SR Ca^2+ ^depletion, mitochondrial Ca^2+ ^uptake and cardiomyocyte apoptosis during hypoxia/reoxygenation (H/Re).

## Materials and methods

### Isolation of neonatal rat cardiomyocytes and H/Re experiments

Primary cultures of neonatal rat cardiomyocytes were performed as previously described [12]. Newborn Wistar rats (1-3 days) were used for this study. The rats were handled in accordance with the Guide for the Care and Use of Laboratory Animals published by the China National Institutes of Health. Briefly, hearts from male Wistar rats (1-3 days old) were minced and dissociated with 0.25% trypsin. Dispersed cells were seeded at 2 × 10^5 ^cells/cm^2 ^in 60-mm culture dishes with Dulbecco's modified Eagle medium (DMEM) supplemented with 10% fetal bovine serum (FBS) and then cultured in a 5% CO_2 _incubator at 37°C. Hypoxic conditions were produced using D-Hanks solution (mM: 5.37 KCl, 0.44 KH_2_PO_4_, 136.89 NaCl, 4.166 NaHCO_3_, 0.338 Na_2_HPO_4_, 5 D-glucose, pH 7.3-7.4 at 37°C) saturated with 95% N_2 _and 5% CO_2_. The pH was adjusted to 6.8 with lactate to mimic ischemic conditions. The dishes were put into a hypoxic incubator that was equilibrated with 1% O_2_/5%CO_2_/94%N_2_. After hypoxic treatment, the culture medium was rapidly replaced with fresh DMEM with 10% FBS (10% FBS/DMEM) to initiate reoxygenation [13].

### Experimental protocols

At 72 h post-culturing with 10% FBS/DMEM, the cells were randomly divided into six groups: (1) control group: cells were continuously cultured for 9 h with 10% FBS-DMEM; (2) H/Re: cells were placed in hypoxic culture medium for 3 h and then reoxygenated for 6 h by replacing hypoxic culture medium with fresh DMEM containing 10% FBS; (3) CaCl_2 _+ NiCl_2 _+ CdCl_2_-H/Re (Ca + Ni + Cd-H/Re): neonatal rat cardiomyocytes were treated with CaCl_2 _(2.2 mM), NiCl_2 _(1 mM) and CdCl_2 _(200 μM) for 30 min in hypoxic medium and then reoxygenated for 6 h by replacing hypoxic culture medium with fresh DMEM containing 10% FBS (CaCl_2 _is an activator of CaR, NiCl_2 _is an inhibitor of the Na^+^-Ca^2+ ^exchanger, CdCl_2 _is a inhibitor of the L-type calcium channel; these drugs do not affect cardiomyocyte viability); (4) NPS-2390 + CaCl_2 _+ NiCl_2 _+ CdCl_2_-H/Re (NPS-2390 + Ca + Ni + Cd-H/Re): neonatal rat cardiomyocytes were treated with NPS-2390 (10 μM) for 40 min, and the following steps were the same as for group 3 (NPS-2390 is an allosteric antagonist of the group 1 metabotropic glutamate receptors); (5) 2-APB + CaCl_2 _+ NiCl_2 _+ CdCl_2_-H/Re (2-APB + Ca + Ni + Cd-H/Re): neonatal rat cardiomyocytes were treated with 2-APB (3 μM) for 40 min, and then other steps were the same as in group 3 (2-APB or 2- aminoethoxydiphenyl borate is a membrane permeable IP_3_R inhibitor); (6) Ruthenium red + CaCl_2 _+ NiCl_2 _+ CdCl_2_-H/Re (Ru + Ca + Ni + Cd-H/Re): neonatal rat cardiomyocytes were treated with Ruthenium red (10 μM) for 40 min, and then underwent the same steps as in group 3 (Ruthenium red is an inhibitor of mitochondrial calcium uniporter ).

### Immunocytochemistry

Cardiomyocytes were fixed in 10% formaldehyde in phosphate-buffered saline (PBS) for 10 min, permeabilized with 0.1% Triton X-100, washed three times in PBS and blocked in PBS containing 5% bovine serum albumin, 5% horse serum and 0.05% Triton X-100 for 1 h at room temperature (RT). Specific subtype anti-IP_3_R rabbit polyclonal antibodies were incubated overnight at 4°C at 1:200 or 1:100 (Santa Cruz). FITC-conjugated anti-rabbit IgG was used as a secondary antibody. As indicated, some cells were stained with 4-6- diamidino-2-phenylindole (25 μg/ml) (DAPI, Roche) for 1 h. The results of immunocytochemical staining were read and recorded with a laser confocal scanning microscope (Olympus, LSM, Japan).

### 3-(4,5-dimethyl thiazol-2yl)-2,5-diphenyltetrazolium bromide(MTT) assay

In the current study, cardiomyocytes were planted in 96-well plates. The MTT assay was performed as described previously [10]. Briefly, MTT (Sigma) was added into the cell cultures at a final concentration of 0.5 mg/mL and the mixture was incubated for 4 h at 37°C. Subsequently, the culture medium was removed and DMSO was added to each well to dissolve the resulting formazan crystals. The absorbance was measured at a wavelength of 570 nm using a microplate reader (Bio-Tek Instruments Inc., Richmond, Va). Background absorbance of medium in the absence of cells was subtracted [14]. Percent viability was defined as the relative absorbance of treated versus untreated control cells.

### Hoechst staining

Apoptotic cells were identified by the distinctive condensed or fragmented nuclear structure in cells stained with the chromatin dye Hoechst 33342 (Sigma). Cells were fixed with 4% paraformaldehyde for 10 min at room temperature and were washed twice with phosphate buffer solution (PBS). Cells were then incubated with 5 μg/mL Hoechst 33342 for 15 min. Next, the cells were washed three times and photographed using fluorescence microscope (Leica DFC500 System; Leica Microsystems, Bannockburn, Ill). At least 500 nuclei from randomly selected fields in each group were analyzed for each experiment, and the percentage of apoptotic cells was calculated as the ratio of the number of apoptotic cells versus the total cells counted.

### Neonatal rat cardiomyocytes loaded with Fluo-4 AM, Fluo-5N AM and X-rhod-1 AM and cell permeabilization

[Ca^2+^]i was determined as previously described [15]. Briefly, cells were seeded on the culture slides. After experimentation, cells were loaded with fluo-4 AM in 1% working solution at 37°C for 1 h, washed three times with Ca^2+^-free PBS to remove extracellular fluo-4 AM, and diluted to the required concentration. The reagents were added in Ca^2+^-free solution (145 mM NaCl, 5 mM KCl, 1.0 mM EGTA, 1 mM MgCl_2_, 10 mM HEPES-Na, 5.6 mM glucose, pH 7.4). Fluorescence measurement of Ca^2+ ^was performed using a laser confocal scanning microscope (Olympus, LSM, Japan) at an excitation wavelength of 485 nm for [Ca^2+^]i and an emission wavelength of 530 nm for [Ca^2+^]i, using the equation [Ca^2+^]i = *K*_d_[(F -F_min_)/(F_max _- F)], where *K*d is the dissociation constant (345 nM for fluo-4), F is the fluorescence at intermediate Ca^2+ ^levels (corrected from background fluorescence), Fmin is the fluorescence intensity of the indicator in the absence of Ca^2+^and is obtained by adding a solution of 10 mM EGTA for 15 min, and F_max _is the fluorescence of the Ca^2+^-saturated indicator and is obtained by adding a solution of 25 μM digitonin in 2.2 nM CaCl_2 _for 15 min. Final values for [Ca^2+^]i are expressed in nanomoles.

To determine [Ca^2+^]_SR_, cardiomyocytes were treated with Fluo-5N acetoxymethylester (10 μM) for 2 h and deesterified for 1.5 h. For intact myocytes, the superfusate contained (in mM) 140 NaCl, 4 KCl, 1 MgCl_2_, 2 CaCl_2_, 10 HEPES, and 10 glucose (pH 7.4, 23°C). For permeabilization, myocytes were exposed to solution (in mM: 0.1 EGTA, 10 HEPES, 120 K-aspartate, 1 free MgCl_2_, 5 ATP, 10 reduced glutathione, and 5 phosphocreatine; pH 7.4) and then permeabilized using saponin (50 μg/ml) for 20 seconds. Excitation was set at 488 nm and emission was measured at 530 nm at room temperature [15]. Images of fluorescence reflecting [Ca^2+^]_i _and [Ca^2+^]_SR _were recorded using a laser confocal scanning microscope (Olympus, LSM, Japan). There were more than 10 cells to be analyzed in each view and quantified using the analysis software for the microscope.

Recent study showed that the mitochondrial Ca^2+ ^concentration ([Ca^2+^]_m_) consistently increases during reoxygenation [12]. Therefore, [Ca^2+^]_m _was measured at 60 min post-reoxygenation. [Ca^2+^]_m _was determined according to the manufacturer's instructions (Molecular Probes). In brief, the cultured cardiomyocytes (1 × 10^6 ^cells/sample) were initially washed with HEPES buffer containing (in mM) 130 NaCl, 4.7 KCl, 1.2 MgSO_4_, 1.2 KH_2_PO_4_, 10 HEPES, 11 glucose, and 0.2 CaCl_2 _at pH 7.4 and then stained with 5 μmol/L X-rhod-1 AM for 30 min at room temperature. To avoid deesterification of intracellular X-rhod-1 AM in the cytosolic compartment, which would interfere with the detection of [Ca^2+^]_m_, the cardiomyocytes were rinsed and incubated with 100 μM MnCl_2_-HEPES for an additional 20 min to quench the cytosolic Ca^2+ ^signal [16]. Fluorescence measurement was determined using a fluorescence plate reader (CytoFluor II; PerSeptive Biosystems; Framingham, MA) at an excitation wavelength of 580 nm and an emission wavelength of 645 nm for [Ca^2+^]_m_. To validate the measurement of [Ca^2+^]_m_, the cultured cardiomyocytes were transferred into a slide chamber after X-rhod-1 AM staining and were placed on the stage of a fluorescence microscope (×50 objective; Olympus). The images from the slides were captured using a digital camera connected to Image-Pro Plus software (Media Cybernetics; Silver Spring, MD). There were more than 10 cells to be analyzed in each view.

### Measurement of mitochondrial membrane potential

Mitochondrial membrane potential (△ψ_m_) was measured with a unique cationic dye of 5,5',6,6'-tetrachloro 1,1'3,3'-tetraethylbenzimidazolcarbocyanine iodide (JC-1), as previously described [12]. Briefly, cells were seeded on culture slides and treated according to experimental protocols. Previous data demonstrated that [Ca^2+^]_m _might continuously increase during the process of reoxygenation and result in mitochondrial △ψ_m _collapse [12], so we detected △ψ_m _at 1 h after reoxygenation. At the end of the above-described treatments, cells were stained with JC-1 (1 μg/ml) at 37°C for 15 min and then rinsed three times with PBS. Observations were immediately made using a laser confocal scanning microscope. In live cells, the mitochondria appear red due to the aggregation of accumulated JC-1, which has absorption/emission maxima of 585/590 nm (red). In apoptotic and dead cells, the dye remains in its monomeric form, which has absorption/emission maxima of 510/530 nm (green). More than 100 areas were selected from each image. The average intensity of red and green fluorescence was determined. The ratio of JC-1 aggregate (red) to monomer (green) intensity was calculated. A decrease in this ratio was interpreted as a decrease in the △ψ_m_, whereas an increase in this ratio was interpreted as a gain in the △ψ_m_.

### Identification of bax/bak translocation to the mitochondria and assay for cytochrome *c *release from mitochondria

Western blotting of cellular fractions was used to quantify changes in cytochrome *c*, bax and bak distribution within cells, as previously described [17]. Briefly, 1 × 10^7 ^rat cardiomyocytes were homogenized in ice-cold Tris-sucrose buffer (in mM: 350 sucrose, 10 Tris-HCl, 1 ethylenediaminetetraacetic acid, 0.5 dithiothreitol, and 0.1 phenylmethanesulfonylfluoride; pH 7.5). After 10 min of incubation, cardiomyocyte homogenates were initially centrifuged at 1000 × g for 5 min at 4°C, and the supernatant was further centrifuged at 40,000 × g for another 30 min at 4°C. The supernatant was saved as the cytosolic fraction. The precipitate was re-suspended in the above buffer (containing 0.5% v/v Nonidet P-40) and saved as the mitochondrial fraction. The mitochondrial fractions were blotted with a primary rat anti-bax, bak and cytochrome *c *monoclonal antibody (Santa Cruz Inc.). The volume of specific bands was measured using a Bio-Rad Chemi EQ densitometer and Bio-Rad QuantityOne software (Bio-Rad laboratories, Hercules, USA).

### Western blotting

Western blot analyses were performed as previously described [18]. In brief, the protein concentration of samples was first determined using the Bio-Rad DC protein assay kit (Bio-Rad Laboratories, Hercules, CA). A total of 20 μg of protein was electrophoresed on a 12% SDS-polyacrylamide gel and transferred to nitrocellulose membranes (Amersham International, Amersham, UK). The membranes were blocked with 10% skim milk in TBST buffer (10 mM Tris, pH 7.6, 150 mM NaCl, and 0.1% Tween 20) for 1 h at room temperature and then incubated with a rabbit anti-BAP31 polyclonal antibody (1:500 dilution, sc-48766, Santa Cruz Biotechnology) overnight at 4°C. HRP-conjugated anti-rabbit IgG (1:3000 dilution, Bio-Rad Laboratories) was used as a secondary antibody. Specific bands were visualized with a chemiluminescent substrate (ECL kit, Amersham International).

### Statistical analyses

Significance was evaluated using student's *t*-test, and p < 0.05 was considered statistically significant. Data are expressed as mean ± standard error of the mean (S.E.M.) and are representative of at least three independent experiments. [Ca^2+^]_i _data were obtained from 2-3 experiments, and 10-12 images were analyzed in each group.

## Results

### Asymmetric subcellular distribution of IP_3_R subtypes in cardiomyocytes

**Figure 1:**
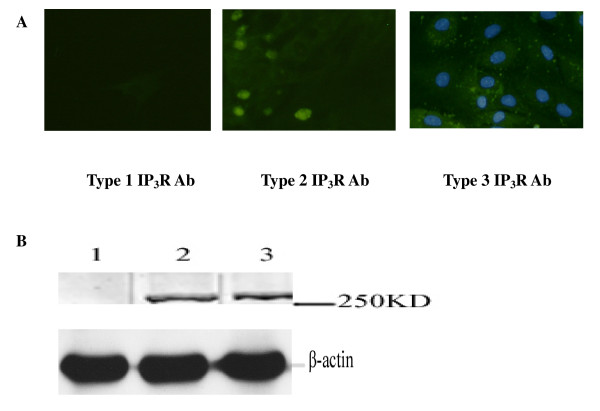
**Subcellular IP**_**3**_**Rs localization**. (A) Immunocytochemical staining of cardiomyocyte with specific antibodies for type 1, type 2 and type 3 IP3Rs. (B) Western blot analysis of cardiomyocyte lysates using antibodies specific for IP3R, type 1, type 2 and type 3, respectively. DAPI and FITC to co-stain nuclei and type 3 IP_3 _receptors and show the spatial relation between the two structures.

Western blot results showed that type 2 and 3 IP_3_Rs were expressed in cardiomyocytes, while type 1 IP_3_R expression was undetectable (Fig. 1A). Similar to the results of the Western blot analysis, type 3 IP_3_R was distributed in the cytoplasm and intense perinuclear and intranuclear staining was evident for type 2 IP_3_R in immunofluorescence study, while type 1 IP_3_R was undetectable.

### Activation of CaR induces cardiomyocyte apoptosis by H/Re

**Figure 2:**
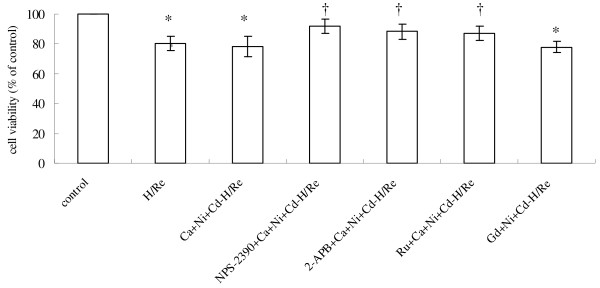
**Viability of cardiomyocytes was examined using the MTT assay**. The cell viability of the control was adjusted to 100%. The data presented are expressed as the mean **± **SEM. *p < 0.05 vs Control group; †p < 0.05 vs Ca + Ni + Cd-H/Re .The experiment was repeated three times with similar results.

To confirm the role of CaR in cardiomyocyte apoptosis evoked by H/Re, we examined whether activation of CaR induced apoptosis in cultured cardiomyocytes of neonatal rats under our experimental conditions. We used two CaR agonists, CaCl_2 _and GdCl_3_, to demonstrate the role of CaR in the induction of apoptosis during H/Re. When cardiomyocytes were exposed to the activation of CaR by H/Re, cell viability was shown to be reduced to 80.2 ± 4.8% (H/Re), 78.3 ± 6.8% (Ca + Ni + Cd-H/Re) and 77.6 ± 5.1% (Gd + Ni + Cd-H/Re), respectively, compared with that of control cells using the MTT assay. Cell viability in NPS-2390 + Ca + Ni + Cd-H/Re (91.7 ± 4.6%), NPS-2390 is an allosteric antagonist of group 1 metabotropic glutamate receptors. 2-APB + Ca + Ni + Cd-H/Re (88.3 ± 5.2%, 2-APB is a selective inhibitor) and Ru + Ca + Ni + Cd-H/Re (87.6 ± 5.6%, Ruthenium red is an inhibitor of mitochondrial calcium uniporter) groups was more than that of the H/Re, Ca + Ni + Cd-H/Re and Gd + Ni + Cd-H/Re groups (Fig. 2).

**Figure 3:**
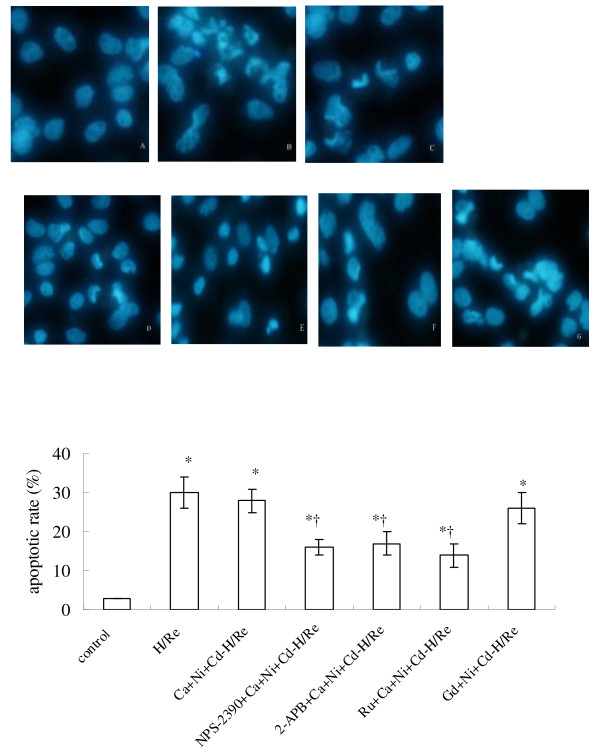
**Hoechst-stained nuclei of apoptotic myocytes were analyzed morphologically and were expressed as the percentage of total nuclei**. (magnification × 400). A: control group. B: H/Re group. C: Ca + Ni + Cd-H/Re group. D: NPS-2390 + Ca + Ni + Cd-H/Re. E: 2-APB + Ca + Ni + Cd-H/Re. F: Ru + Ca + Ni + Cd-H/Re group. G: Gd + Ca + Ni + Cd-H/Re The cardiomyocytes were placed in hypoxic culture medium for 3 h and then reoxygenated for 6 h by replacing hypoxic culture medium with fresh DMEM containing 10% FBS, and were treated with different inhibitors, respectively. The data presented are expressed as the mean **± **SEM. *p < 0.05 vs Control group; †p < 0.05 vs Ca + Ni + Cd-H/Re.

To further determine whether the cell death induced by H/Re and activation of CaR was mediated by apoptosis, the nuclear morphology was analyzed using the Hoechst staining assay. The apoptotic cells exhibited typical fragmented nuclei and condensed chromatin on staining with Hoechst 33342 (Fig. 3). The percentage of apoptotic cells relative to the total number of cells was increased to H/Re (33 ± 6%), Ca + Ni + Cd-H/Re (31 ± 5%) and Gd + Ni + Cd-H/Re (34 ± 3%) compared with the NPS-2390 + Ca + Ni + Cd-H/Re (20 ± 4%), 2-APB + Ca + Ni + Cd-H/Re (18 ± 4%) and Ru + Ca + Ni + Cd-H/Re (23 ± 5%) groups. Therefore, these data show that the activation of CaR is involved in H/Re - induced cardiomyocyte apoptosis.

### CaR-mediated Ca^2+ ^release in cardiomyocytes during hypoxia/reoxygenation

**Figure 4:**
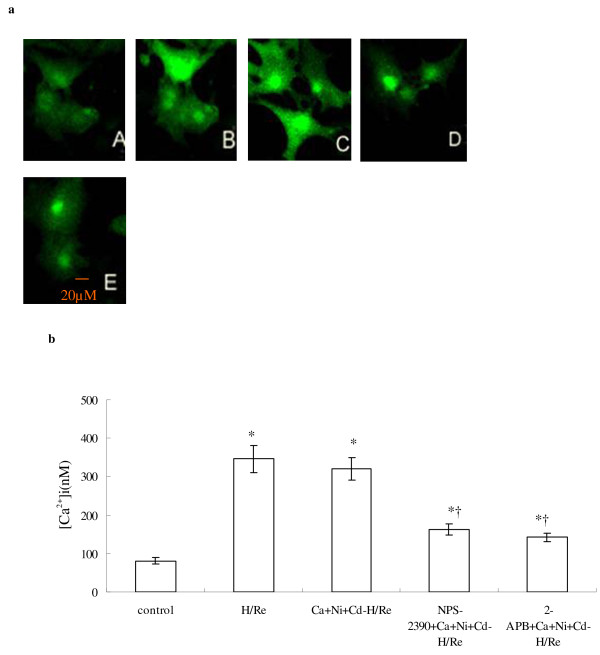
**The measurement of [Ca**^**2+**^**] after hypoxia/reoxygenation by laser confocal microscopy**. (a) A: Control group. B: H/Re group. C: Ca + Ni + Cd-H/Re group. D: NPS-2390 + Ca + Ni + Cd-H/Re. E:2-APB + Ca + Ni + Cd-H/Re -H/Re. (b) Values represent the group mean ± SEM of at least four independent experiments. *p < 0.05 vs Control group; †p < 0.05 vs Ca + Ni + Cd-H/Re.

According to previous reports, the increase of [Ca^2+^]i in cardiomyocytes occurs in the early phase of reoxygenation, concomitant with the burst of calcium overload [19]. In our study, we quantified [Ca^2+^]i during the first hour after reoxygenation. [Ca^2+^]i was measured by fluo-4 AM staining (sensitive Ca^2+ ^probe). The calcium concentration of the H/Re (346 ± 35 nM) and Ca + Ni + Cd-H/Re (321 ± 29 nM) groups was significantly increased compared to the control (81 ± 9 nM), NPS-2390 + Ca + Ni + Cd-H/Re (163 ± 15 nM) and 2-APB + Ca + Ni + Cd-H/Re (142 ± 11 nM) groups (Fig.4). The CaCl_2 _-induced increase in intracellular calcium was significantly attenuated by NPS-2390, which was shown previously to modulate the effects of Ca^2+ ^in other CaR-expressing cells [16]. In our study, we also found similar results in neonatal cardiomyocytes. Likewise, the CaCl_2_-induced increase in [Ca^2+^]i was also significantly reduced by 2-APB compared to the Ca + Ni + Cd-H/Re group (Fig. 4).These results suggest that CaCl_2 _may activate CaR that then induces Ca^2+ ^release through a PLC-mediated/IP_3_-dependent process.

### Activation of CaR depletes [Ca^2+^]_SR _during H/Re

**Figure 5:**
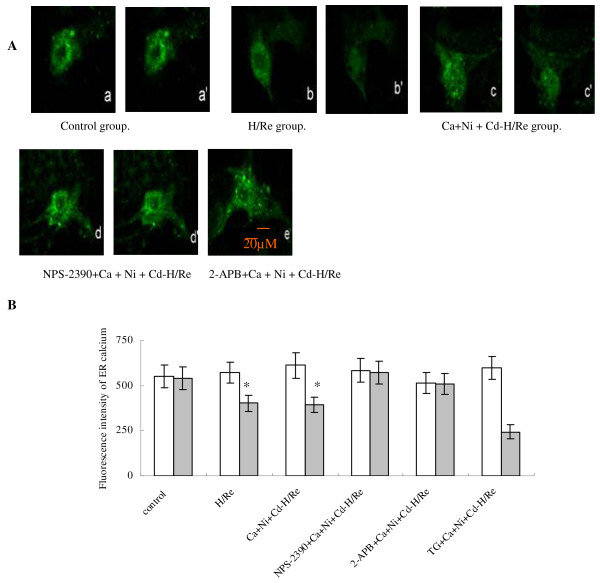
**CaR activation induced Ca**^**2+ **^**release from the ER during H/Re**. (A) a images represent the beginning of reperfusion (0 min). a' images represent 60 min after reperfusion. (B) Values represent the group mean **± **SEM of at least four independent experiments. *p < 0.05 vs Control group; †p < 0.05 vs Ca + Ni + Cd-H/Re . White bar represents reoxygenation 0 min; grey bar represents reoxygenation 60 min.

We have demonstrated that CaCl_2_-activated CaR induces the increase of [Ca^2+^]i, but the origin of intracellular calcium remains unclear. We examined [Ca^2+^]_SR _by Fluo-5N staining. Fluo-5N is a low-affinity Ca^2+ ^indicator (K_d _= 400 μmol/L) that is only bright where [Ca^2+^] is very high, such as in the SR [15]. Rat neonatal cardiomyocytes were loaded with Fluo-5N and permeabilized with saponin. Irregularly distributed bright spots were seen in cardiomyocytes. The Fluo-5N signal was stable at the beginning of reperfusion (Fig. 5). At 60 min after reperfusion, the Fluo-5N signal was detected in the SR. We found that the fluorescence intensity in the SR in the Ca + Ni + Cd-H/Re (376 ± 44) and H/Re (399 ± 42) groups was significantly decreased compared to the control (648 ± 62), NPS-2390 + Ca + Ni + Cd-H/Re (562 ± 64) and 2-APB + Ca + Ni + Cd-H/Re (532 ± 51) groups. Luo et al. have previously demonstrated that 3 μM 2-APB inhibited IP_3_Rs and prevented PE-induced enhancement of Ca^2+ ^sparks in neonatal cardiomyocytes [20]. Our study also suggests that 3 μM 2-APB may decrease [Ca^2+^]i through the inhibition of Ca^2+ ^release from the SR via IP_3_R. Thus, 2-APB treatment could maintain the fluorescence intensity in the SR of cardiomyocytes during reperfusion. These results suggested that the activation of CaR by CaCl_2 _or H/Re induced SR release of Ca^2+^.

### Activation of CaR increases [Ca^2+^]_m _and reduces the mitochondrial membrane potential

**Figure 6:**
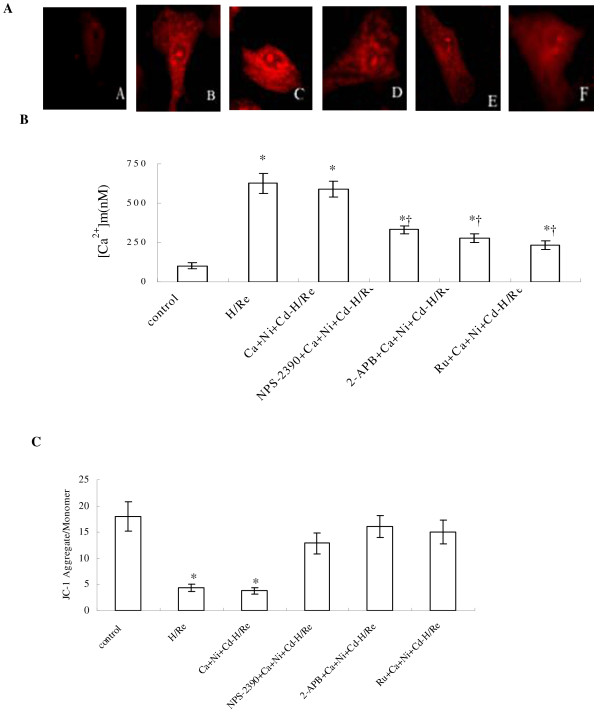
**The measurement of [Ca^2+^]m after 1 h of reoxygenation by laser confocal microscopy**. A: control group. B: H/Re group. C: Ca + Ni + Cd-H/Re group. D: NPS-2390 + Ca + Ni + Cd-H/Re E: 2-APB + Ca + Ni + Cd-H/Re -H/Re. F: Ru + Ca + Ni + Cd-H/Re group. (B) Value represents the group mean ± SEM of at least four independent experiments. *p < 0.05 vs Control group; †p < 0.05 vs Ca + Ni + Cd-H/Re . (C) Effect of hypoxia/reoxygenation and CaR activation on △ψm in neonatal rat cardiomyocytes Summarized data for the relative changes of JC-1 fluorescence. Data are mean ± SEM. †p < 0.05 vs sham control group *p < 0.05 vs Ca + Ni + Cd-H/Re group.

Although CaCl_2_-activated CaR significantly reduced [Ca^2+^]_SR_, the role of type 3 IP_3_Rs at the MAM in mediating Ca^2+ ^uptake to mitochondria is less clear. To address this question, [Ca^2+^]_m _was measured at 60 minutes post-reoxygenation by X-rhod-1 AM staining. The [Ca^2+^]_m _was markedly low in the control group (108 ± 11 nM, Fig. 6.A). The [Ca^2+^]_m _was significantly greater in the H/Re (626 ± 65 nM) and Ca + Ni + Cd-H/Re (589 ± 52 nM) groups than in the NPS-2390 + Ca + Ni + Cd-H/Re (331 ± 27 nM), 2-APB + Ca + Ni + Cd-H/Re (277 ± 29 nM), or Ru + Ca + Ni + Cd-H/Re (233 ± 26 nM)groups.

The mitochondrial membrane potential was detected with JC-1 staining (Fig. 6C). The ratio of JC-1 aggregates (red) to monomer (green) intensity was reduced in the H/Re (4.4 ± 0.7) and Ca + Ni + Cd-H/Re (3.8 ± 0.6) groups compared with the control (18.1 ± 3.2), NPS-2390 + Ca + Ni + Cd-H/Re (12.9 ± 2.7), 2-APB + Ca + Ni + Cd-H/Re (16.4 ± 2.1) and Ru + Ca + Ni + Cd-H/Re (15.5 ± 2.4) groups.

### [Ca^2+^]_SR _depletion induced by CaR activation causes apoptosis via a mitochondria-mediated pathway

**Figure 7:**
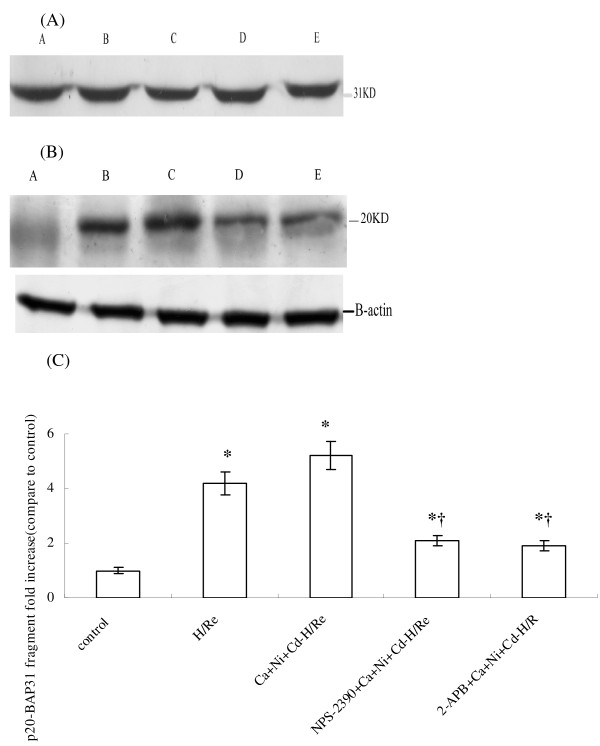
**The intact (A) and p20 (B) of BAP31 expression during H/Re**. A: sham control group. B: H/Re group. C: Ca + Ni + Cd-H/Re group. D: NPS-2390 + Ca + Ni + Cd-H/Re. E: 2-APB + Ca + Ni + Cd-H/Re. The fold change values were mean ± SEM n = 3-4.*p < 0.05 vs control group †p < 0.05 vs H/Re (C)

BAP31, an integral membrane protein of the SR, is a caspase-8 substrate [21]. It is cleaved into a p20 fragment following CaCl_2 _treatment during H/Re (Fig.7). The p20 fragment expression was higher in the H/Re (4.57 ± 0.42) and Ca + Ni + Cd-H/Re (5.28 ± 0.59) groups than in the NPS-2390-+Ca + Ni + Cd-H/Re (2.16 ± 0.27) and 2-APB + Ca + Ni + Cd-H/Re (1.94 ± 0.21) groups.

**Figure 8:**
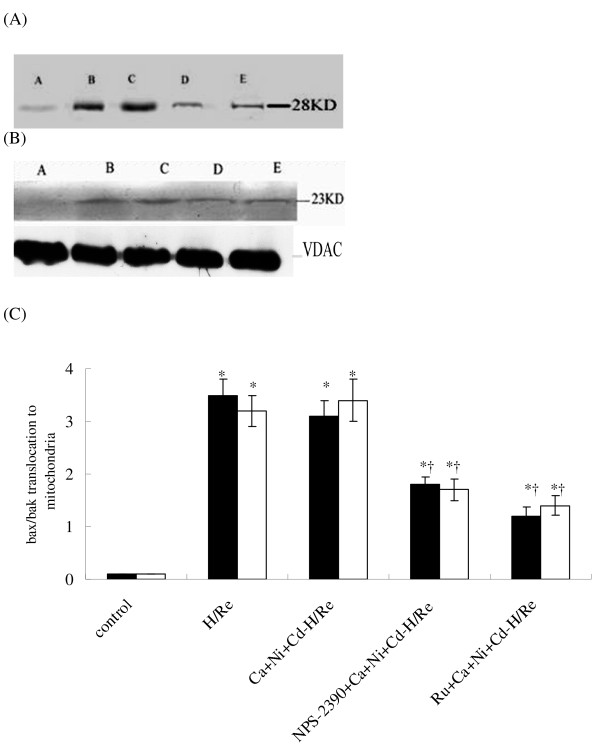
**Bax (A) and bak (B) translocation to the mitochondrial fractions in rat cardiomyocytes after H/Re**. A: control group, B: H/Re group, C: Ca + Ni + Cd-H/Re group, D: NPS-2390 + Ca + Ni + Cd-H/Re group and E: Ru + Ca + Ni + Cd-H/Re group. The fold-change values are mean ± SEM, n = 3-4, *p < 0.05 vs. control group †p < 0.05 vs. H/Re (C). Black bar represented the fold change of bax; white bar represented the fold change of bak.

**Figure 9:**
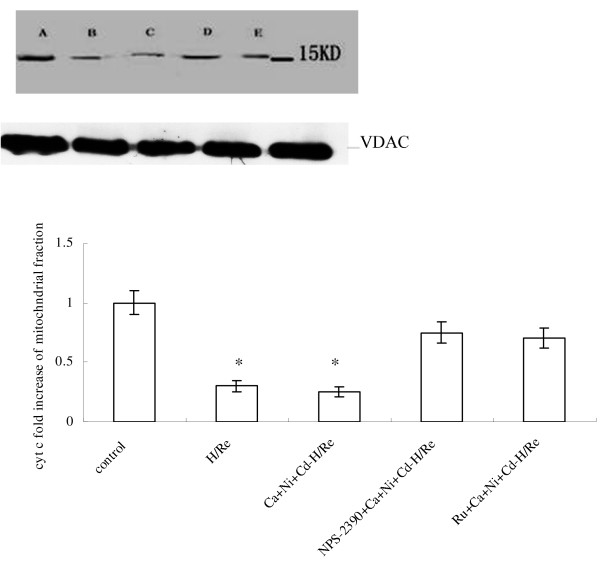
**The release of cytochrome-C from mitochondrial fractions**. A: control group. B: H/Re group. C: Ca + Ni + Cd-H/Re group. D: NPS-2390 + Ca + Ni + Cd-H/Re group. E: Ru + Ca + Ni + Cd-H/Re group. The fold change of cyt c values are mean ± SEM n = 3-4. *p < 0.05 vs control group †p < 0.05 vs H/Re.

The p20-BAP31 protein has been shown to direct pro-apoptotic signals between the SR and the mitochondria, resulting in the insertion of bax and bak into the outer mitochondria membrane, homo-oligomerization and release of cyt c from the mitochondria [22]. Our results suggest that bax and bak translocation to the mitochondria was significantly increased in the H/Re (3.52 ± 0.31, 3.22 ± 0.28) and Ca + Ni + Cd-H/Re (3.16 ± 0.33, 3.44 ± 0.41) groups compared with the NPS-2390 + Ca + Ni + Cd-H/Re (1.86 ± 0.15, 1.77 ± 0.22) and Ru + Ca + Ni + Cd-H/Re (1.29 ± 0.17, 1.4 ± 0.18) groups (Fig. 8). Next, mitochondrial release of cytochrome *c *was analyzed to prove the role of the mitochondrial apoptotic pathway. It was found that cytochrome *c *from mitochondria in the H/Re (0.3 ± 0.05) and Ca + Ni + Cd-H/Re (0.25 ± 0.04) groups was significantly decreased compared with the control (1.0 ± 0.1), NPS-2390- + Ca + Ni + Cd-H/Re (0.75 ± 0.09) and Ru + Ca + Ni + Cd-H/Re (0.69 ± 0.08) groups (Fig. 9).

## Discussion

This study was designed to address the potential involvement of the sarcoplasmic reticulum and mitochondria in regulating cardiomyocyte Ca^2+ ^signaling through MAM subjected to CaR activation and H/Re. The main findings of this study are as follows: (i) Activation of CaR induced the release of Ca^2+ ^from the SR and, simultaneously, the increase of Ca^2+ ^uptake into the mitochondria through MAM during H/Re. (ii) The CaR activation increased the expression of the p20-BAP31 fragment, the translocation of bax/bak from the cytoplasm to the mitochondria and the release of cytochrome *c *from the mitochondria during H/Re.

The membrane receptor CaR couples to the enzyme PLC, which liberates IP_3 _from phosphatidylinositol 4,5-bisphosphate (PIP_2_). The major function of IP_3 _is to induce endogenous Ca^2+ ^release through IP_3_Rs [23]. Ca^2+ ^is the primary agonist of CaRs. The EC50 for Ca^2+ ^activation of the CaR is 3-4 mM [24]. CaCl_2 _was chosen as an agonist to activate CaR, and was shown to increase the expression of CaR (Additional file 1). NPS-2390 was chosen as an antagonist of CaR. In previous study, NPS-2390 is an allosteric antagonist of the group 1 metabotropic glutamate receptors. Group 1 metabotropic glutamate receptors are seven transmembrane domain G protein coupled receptors that activate the Gaq class of G-proteins and stimulate Phospholipase C, resulting in phosphoinositide(PI) hydrolysis and the formation of inositol triphosphate and diacylglycerol.

IP_3_Rs are ligand-gated Ca^2+ ^channels that function to release intracellular Ca^2+ ^(predominantly from the sarcoplasmic reticulum) in response to IP_3 _[5]. During reoxygenation, CaR activation caused a significant decrease in the [Ca^2+^]_SR_, which could be reversed by either the CaR inhibitor NPS-2390 or the IP_3_Rs inhibitor 2-APB. Furthermore, the type 3 isoform of the IP_3_R localized to the SR membranes. Taken together, these results suggest that activation of CaR is involved in the release of Ca^2+ ^from the SR through the IP_3_R during H/Re.

Rizzuto et al. have provided a structural basis for this hypothesis by showing that mitochondria and ER form an interconnected network in living cells with a restricted number of close contacts [25]. It has been reported that IP_3_Rs play an important role in establishing macromolecular complexes on the surface of the SR membranes and in modulating the linkage between the SR and mitochondrial membranes. Mitochondria respond rapidly to physiological increases in [Ca^2+^]e, and stimulation with Gq-coupled receptor agonists, which induce IP_3 _production and the subsequent release of Ca^2+ ^from ER, causes a rapid rise in [Ca^2+^]_m _[26]. This effect has been detected in many cells types: HeLa cells, fibroblasts, endothelial and epithelial cells, cardiac and skeletal muscle cells, neurons and pancreatic β cells [27,28]. CaR, as a Gq-coupled receptor, could be involved in promoting Ca^2+ ^release from ER and then in induced the [Ca^2+^]_m _rise. Our results suggest that [Ca^2+^]_m _was elevated and mitochondrial membrane potential collapsed in the Ca + Ni + Cd-H/Re group, whereas [Ca^2+^]m and mitochondrial membrane potentials were maintained in the 2-APB + Ca + Ni + Cd-H/Re group. The rapid mitochondrial Ca^2+ ^uptake is related to the low affinity of the Ca^2+ ^transport system. Therefore, Ruthenium red, an inhibitor of the mitochondrial calcium transporter, was used in our experiment. The results reveal that [Ca^2+^]_m _and mitochondrial potentials were maintained in the Ru + Ca + Ni + Cd-H/Re group. These results suggest that both the SR and the mitochondria orchestrate the regulation of Ca^2+ ^signaling between these two organelles.

Although a role for the SR in the mitochondrial redistribution of Ca^2+ ^has been implicated in many models of apoptosis, a primary role for IP_3 _generation and the activation of IP_3_Rs in this process has been examined in only a few instances. Caspase-8 cleavage of BAP31 at the SR leads to the generation of a p20 fragment, which directs pro-apoptotic signals between the SR and mitochondria, resulting in early discharge of Ca^2+ ^from the SR and its concomitant uptake into the mitochondria. Early and critical events in apoptosis occur in mitochondria and in the ER, and the release of elements acting as caspase cofactors, such as cytochrome c (from mitochondria) and Ca^2+ ^(from the ER), into the cytosol are requisites for cell death in many cases [29]. The mitochondrial pathway of apoptosis is regulated by members of the Bcl-2 protein family, subdivided into two groups: anti-apoptotic (Bcl-2) and pro-apoptotic (Bax, Bak). The link between Bcl-2 (localized in several intracellular membranes including those of mitochondria and the ER) and Ca^2+ ^homeostasis has been established by showing that Bcl-2 reduces the steady state Ca^2+ ^levels in the ER, thereby dampening the apoptotic signal [30,31]. Jiang et al. showed that CaR was involved in neonatal cardiomyocyte apoptosis in ischemia/reperfusion injury. They suggested that [Ca^2+^]i was increased, inhibiting the expression of Bcl-2 and elevating the expression of the pro-apoptotic protein caspase-3 in cytoplasm [32]. However, the Ca^2+^-dependent model of apoptosis was subsequently supported by a series of observations with the pro-apoptotic Bcl-2 family members Bax and Bak. Cells deriving from knockout mice lacking Bax and Bak that are very resistant to apoptotic death have a dramatic reduction in the [Ca^2+^] within the ER and a drastic reduction in the transfer of Ca^2+ ^from the ER to mitochondria [33].This change prompts mitochondrial fission and cytochrome *c *release into the cytosol. Green et al. demonstrated that [Ca^2+^]_SR _depletion caused bax- and bak-mediated permeability of the outer mitochondrial membrane, thereby releasing pro-apoptotic factors and particularly cytochrome *c *[34]. Our present data show that CaR activation induced the cleavage of BAP31 with the formation of the pro-apoptotic p20 fragment, causing bax and bak translocation to the mitochondria and cytochrome *c *release from the mitochondria during H/Re.

In conclusion, our results constitute the first report that CaR plays an important role in the SR-mitochondrial inter-organelle Ca^2+ ^signaling through the IP_3_Rs, which are also involved in apoptosis during H/Re.

## Abbreviations

IP_3_Rs: inositol 1,4,5-trisphosphate receptors; MAM: mitochondrion-associated ER membrane; H/Re: hypoxia/reoxygenation; CaR: calcium sensing receptor; GPCR: G protein-coupled receptors; PIP_2_: phosphatidylinositol 4,5-bisphosphate; MTT: 3-(4,5-dimethyl thiazol-2yl)-2,5-diphenyltetrazolium bromide; JC-1: 5,5',6,6'-tetrachloro 1,1'3,3'-tetraethylbenzimidazolcarbocyanine iodide

## Competing interests

The authors declare that they have no competing interests.

## Authors' contributions

WZ and CX drafted the manuscript, FL and ZT participated in the design of the study and did most of the experiments, YZ conceived of the study, HL, HR, HZ, CL and GH participated in its design and coordination, YT, BY and RW revised the paper and gave some suggestions. All authors read and approved the final manuscript.

## Supplementary Material

Additional file 1 **CaR inducing apoptosis via the sarcoplasmic reticulum-mitochondrion crosstalk in hypoxia/reoxygenation**. Click here for file
