# Supplementary material for: Calcium-sensing receptors regulate cardiomyocyte Ca2+ signaling via the sarcoplasmic reticulum-mitochondrion interface during hypoxia/reoxygenation
Source: J Biomed Sci. 2010 Jun 17;17(1):50. doi: 10.1186/1423-0127-17-50 (PMC2908572; doi:10.1186/1423-0127-17-50)
Supplement: Additional file 1 — CaR inducing apoptosis via the sarcoplasmic reticulum-mitochondrion crosstalk in hypoxia/reoxygenation. [file 1423-0127-17-50-S1.DOC]

**Additional file 1.** CaR inducing apoptosis via the sarcoplasmic reticulum-mitochondrion crosstalk in hypoxia/reoxygenation.

**1. Materials and methods**

**1.1 Western blot**

Western blot analyses were performed as previously described [14]. Primary antibodies to the CaR were purchased from Santa Cruz, CA, USA. Specific bands were seen after incubation with peroxidase-linked/HRP-labeled secondary antibodies by chemiluminescence using a ECL kit. (Super-enhanced chemiluminescence detection reagents were purchased from Applygen Technologies Inc., Beijing, China.)

**Results**

**1. CaR expression during H/Re**

CaR proteins have a relative molecular mass of 110, 130 and 150 kD. In our study, we detected all the bands of the CaR proteins (Fig. S1). The 130kD band was unclear, so we did not analyze the density of this band. The 150-kD CaR is the mature form of the receptor that is glycosylated with complex carbohydrates. The band at 115–120 kD reflects the cleavage of the NH_2_ terminus from the nucleotide sequence. The 150-kD band of the CaR in the H/Re, Ca + Ni + Cd-H/Re and NPS-2390+Ca+Ni+Cd-H/Re groups was expressed at higher levels compared with the control group.

**B**

**A**


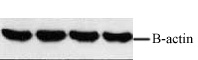


Fig. S1 **(A) Western blot analysis for the CaR in cardiomyocytes during H/Re.** A: control, B: H/Re, C: Ca+Ni + Cd-H/Re, and D: NPS-2390+Ca + Ni + Cd-H/Re group. The control was detected as a basal level of CaR, which served as a baseline, and other groups were compared to control to compute fold-increase. (B) Quantification of western blot analysis for the 150-kD CaR is shown in the lower panel. The fold change values were mean±SEM, n=3–4, *p<0.05 vs. the control group.
